# Karyotypic diversity within the genus *Makalata* (Echimyidae: Echimyinae) of Brazilian Amazon: Chromosomal evidence for multiple species

**DOI:** 10.1371/journal.pone.0235788

**Published:** 2020-07-07

**Authors:** Adenilson Leão Pereira, Stella Miranda Malcher, Cleusa Yoshiko Nagamachi, Augusto César Paes de Souza, Julio Cesar Pieczarka

**Affiliations:** 1 Laboratório de Citogenética, Centro de Estudos Avançados da Biodiversidade, Instituto de Ciências Biológicas, Universidade Federal do Pará, Belém, Pará, Brazil; 2 Instituto Federal do Pará, Abaetetuba, Pará, Brazil; Universita degli Studi di Roma La Sapienza, ITALY

## Abstract

The genus *Makalata* is a taxonomically complex group of rodents on which few cytogenetic studies have been performed. Most of the published karyotypes were described based only on conventional chromosome staining. Here, we studied the karyotypes of *Makalata* from two Brazilian Amazonian states, Amapá and Pará, by Giemsa-staining, G- and C-banding, AgNO3-staining and FISH with 18S rDNA and telomeric sequences probes. We observed 2n = 66/FN = 124 in the Pará state population in *Makalata* sp; and 2n = 72/FN = 128 in the Amapá state population in *M*. *didelphoides*. Multiple chromosome rearrangements may have given rise to these karyotypes, which differ significantly from each other and from those reported in the literature. The chromosomal differences among the described *Makalata* karyotypes can act as a barrier to gene flow; since they are also associated with geographic barriers (e.g., rivers) and numerous molecular differences, they could be seen as evidence for reproductive isolation of populations from genus *Makalata*. Our data suggest that the genus is chromosomally diverse and the karyotypes may belong to different species. These karyotypes may prove useful as taxonomic markers for these rodents.

## Introduction

Echimyidae is considered to be the most ecologically, taxonomically and morphologically diverse family of South American Hystricognathi rodents [1, 2]; there are nine genera and approximately 36 species, and the members are predominantly arboreal [2]. *Makalata* (Husson, 1978) is a genus of Echimyinae that has arboreal and nocturnal habits and has adapted to the floodplain forests (e.g., várzea and igapó); these rodents usually have short legs, long backs, and coarse thorn-shaped fur along their bodies and long tails [3–5]. This genus is distributed from Ecuador, Colombia, Venezuela, the islands of Trinidad and Tobago and Peru, to the Amazon and Brazilian Northeast Region [5–7]. *Makalata* has an unstable taxonomy that has prompted a great deal of disagreement among researchers, who have proposed different compositions and diagnoses for this cryptic group [8]. Some species present phenotypic plasticity while others are morphologically cryptic [8]. The most recent taxonomic compilation recognizes only two species: *M*. *didelphoides* and *M*. *macrura* [5]. *Makalata obscura*, a third species, had its holotype lost and cannot be assigned to a specific population [6]. However, studies based on cytochrome-b haplotypes indicate that *M*. *didelphoides* is composed of at least two well-differentiated geographic units, while *M*. *macrura* is composed of three [4, 9]. In a more recent work [10] cytogenetic and phylogenetic analyses were performed with four molecular markers (cytochrome b, cytochrome oxidase subunit I, exon 28 of the Von Willebrand factor and intron 7 of beta-fibrinogen). These analyses revealed that there are four different clades: two related to *M*. *didelphoides* and two related to *M*. *macrura*. In the largest molecular study performed on this genus to date [8], the authors analyzed karyotypes and cytochrome b sequences and delimited 14 potential species; one was associated with *M*. *macrura* and none was associated with *M*. *didelphoides*, demonstrating that the present taxonomy of this genus does not come close to reflecting its real diversity. In the absence of a complete review of the genus taxonomy, many species remains formally undescribed and are referred to as “*Makalata* sp.”. For clarity, we will herein name the “*Makalata* sp.” taxa by sequential numbering (see Table 1).

**Table 1:** Karyotypes described for genus *Makalata*. See Fig 1 for a map showing the collection locations. Many differentiated geographic units have different karyotypes but have not been formally described as species; they are referred to as “*Makalata* sp.” The letters in the “Karyotype” column are labels for the different karyotypes mentioned herein, and are included to facilitate our discussion of data.

Species	2n	FN	Karyotype	Town or River/State	Reference
*Makalata didelphoides*	66	106	A	Uatumã river, Amazonas state	16
*Makalata* sp. 1	70	120	B	Jamari river, Rondonia state	17*
*Makalata* aff. *macrura*	72	134	C	Barcelos, Amazonas state	10
*Makalata* aff. *didelphoides*	76	134	D	Santa Isabel do Rio Negro, Amazonas state	10
*Makalata didelphoides*	64	100	E	Caracaraí, Roraima state	10
*Makalata didelphoides*	68	132	F	São Valério da Natividade, Tocantins state	10
*Makalata* sp. 2	70	130	G	Juruema and Teles Pires rivers, Mato Grosso state	8
*Makalata macrura*	72	120	H	Lower Madeira river, upper Tapajos river, Para state	8
*Makalata* sp. 3	72	132	I	Trombetas river, Para state	8
*Makalata* sp. 4	70	-	J	Jau river, Amazonas state	8

*Described by Leal-Mesquita (1991, Estudos citogenéticos em dez espécies de roedores brasileiros da família Echimyidae. MSc dissertation. Departamento de Biologia, Instituto de Biociências, Universidade de São Paulo, São Paulo, Brazil) and mentioned by [17] in their Table 1.

**Fig 1:**
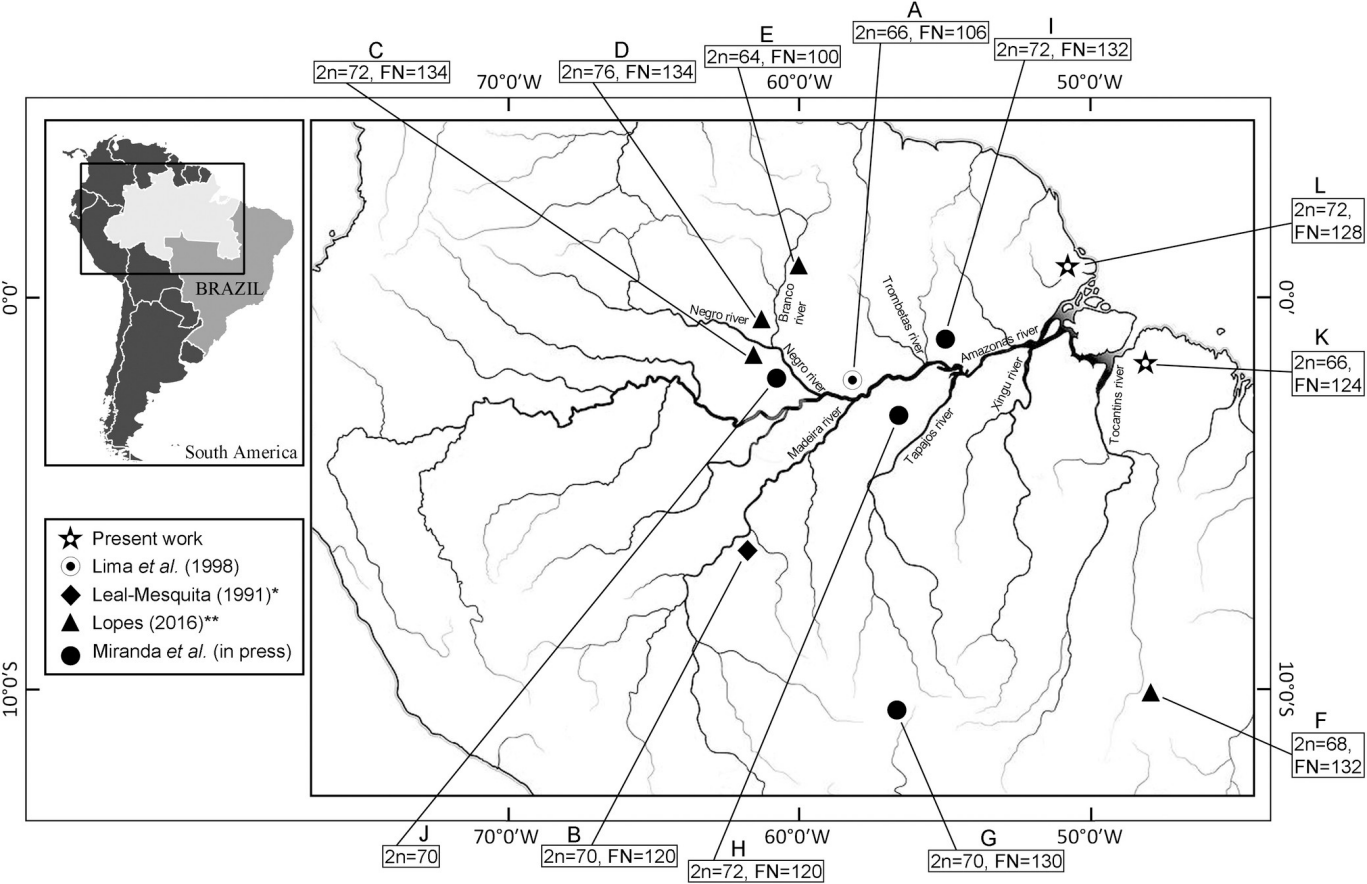
Distribution and sampling locations of *Makalata* species of the Brazilian Amazon region for which karyotypes are available. Leal-Mesquita ERRB (1991)* Estudos citogenéticos em dez espécies de roedores brasileiros da família Echimyidae. Mastership dissertation. Departamento de Biologia, Instituto de Biociências, Universidade de São Paulo, São Paulo, Brazil (mentioned in [17]); Lopes APM (2016)** Estudos citogenéticos, filogenéticos e padrão de distribuição de *Makalata* (Rodentia: Echimyidae) do leste da Amazônia e do nordeste do Cerrado. Rio de Janeiro: Mastership dissertation, Instituto Oswaldo Cruz, Fiocruz [10]. Free map from https://pt.dreamstime.com, image 151248743.

The members of the Echimyidae family exhibit extraordinary chromosomal variation, with the 2n ranging from 14, 16 and 17 in *Proechimys* [11–13] to 118 in *Dactylomys dactylinus* and *Dactylomys boliviensis* [14, 15]. The karyotypic information available for *Makalata* is scarce and incipient, and many karyotypes were described based only on conventional chromosome staining. Lima et al. [16] described a karyotype with 2n = 66/FN = 106 for *M*. *didelphoides* found in Amazonas State, Brazil. In a mastership dissertation, Leal-Mesquita (1991) [17] described a karyotype with 2n = 70/FN = 120 for *Makalata* sp. found in Rondonia State, Brazil, and referred to it as *M*. *armata* [17]. In another mastership dissertation, Lopes [10] described four karyotypes for this genus. A recent publication [8] describe more four karyotypes. Table 1 and Fig 1 show details on the available information; in the former, the karyotypes are given different letters to facilitate our discussion. These studies demonstrate that the genus is chromosomally diverse. Thus, detailed cytogenetic studies are needed to improve our knowledge of the chromosomal evolution of this genus, as well as to assist in the elucidation of its taxonomic relationships.

In the present study, we report two new karyotypes for *Makalata*, from specimens collected in Pará and Amapá states, Eastern Amazon region, Brazil. These karyotypes differ significantly from those in the literature, reinforcing the proposition that *Makalata* is composed of many species.

## Material and methods

### Samples

We analyzed three specimens of *Makalata* sp. 5 (species undefined and numbered as “5” following the criteria of Table 1) collected from the banks of the Sirituba and Caripetuba Rivers in a floodplain area of Abaetetuba town in Pará State, Brazil, and 13 specimens of *M*. *didelphoides* (diagnosed according to [6]), collected from the Araguari River of Porto Grande town in Amapá State, Brazil (S1 Table). The skins and skulls of the analyzed vouchers were deposited at the Museu Paraense Emílio Goeldi (MPEG), Belém City, Pará State, Brazil, and at the Instituto de Pesquisas Científicas e Tecnológicas do Estado do Amapá (IEPA), Macapá City, Amapá State, Brazil. Julio Pieczarka has a permanent field permit (number 13248) from the Instituto Chico Mendes de Conservação da Biodiversidade for collecting samples of Brazilian biodiversity. The Cytogenetics Laboratory of Centro de Estudos Avançados da Biodiversidade, Instituto de Ciências Biológicas, Universidade Federal do Pará, has a special permit (number 19/2003) from the Ministry of Environment for the transport of samples and a permit (number 52/2003) for using the samples in research. The relevant ethics committee (Comitê de Ética Animal da Universidade Federal do Pará) approved this research (Permit 68/2015). The rodents were maintained in the lab with food and water, free from stress, until their euthanasia by intraperitoneal (IP) injection of buffered and diluted barbiturates under local anesthetic.

### Cytogenetic analysis

Each chromosome preparation was obtained by direct extraction of bone marrow, which was performed as previously described [18] with modifications. Briefly, 0.05% colchicine was injected intraperitoneally (0.01 mL/10 g of animal weight). After 40 minutes, the animal was euthanized as described in “Samples”. Bone marrow was extracted from the femur, dipped in a hypotonic solution of potassium chloride (KCl, 0.075M), incubated for 20–30 minutes at 37ºC, centrifuged, resuspended in cold Carnoy’s solution (methanol and glacial acetic acid, 3:1 ratio) and then subjected to a second round of centrifugation and fixative resuspension.

The karyotype was analyzed by conventional staining for 5–8 minutes (Giemsa 5% [Merck], diluted in phosphate buffer, which contained 10.6794 g of Na2HPO4. 2H2O and 8.1654 g of KH2PO4 per 1 liter of distilled water, pH 6.8). For G-banding, fixed metaphases were treated with trypsin, dried, incubated in concentrated saline solution (0.5X SSC) for 5–10 seconds and stained with Wright stain [19]. For C-banding, slides were aged at 37°C for 48 hours, treated in 0.2 N hydrochloric acid (HCl) at room temperature for 30 minutes, washed with distilled water and allowed to dry. Each slide was then immersed in an aqueous solution of barium hydroxide (BaOH 2.5%, filtered) at 60°C for 40–60 seconds, immersed in 0.2 N HCl and washed with distilled water. The slide was incubated in 2x SSC saline solution at 60°C for 30 minutes, washed in distilled water and stained with Wright's solution for 10–15 minutes [20]. For Ag-NO_3_ staining, two drops of colloidal developer solution (2 g of powdered gelatin in 100 mL of deionized water) and 4 drops of aqueous silver nitrate (4 g AgNO3 in 8 ml deionized water) were pipetted onto chromosome preparations, which were then incubated at 60°C for 30 seconds to 2 minutes, washed in distilled water and stained with Giemsa stain [21].

Fluorescence *in situ* hybridization (FISH) with digoxigenin-labeled telomeric probes (All Human Telomere Probes, ONCOR) was performed according to the manufacturer’s protocol. FISH with 18S rDNA probes from *Prochilodus argenteus* was labeled with digoxigenin by nick translation, as previously described [22]. Hybridization signals were detected with Anti-digoxigenin-FITC.

## Results

### *Makalata* sp. 5 (Abaetetuba, Pará, Brazil)

**Fig 2:**
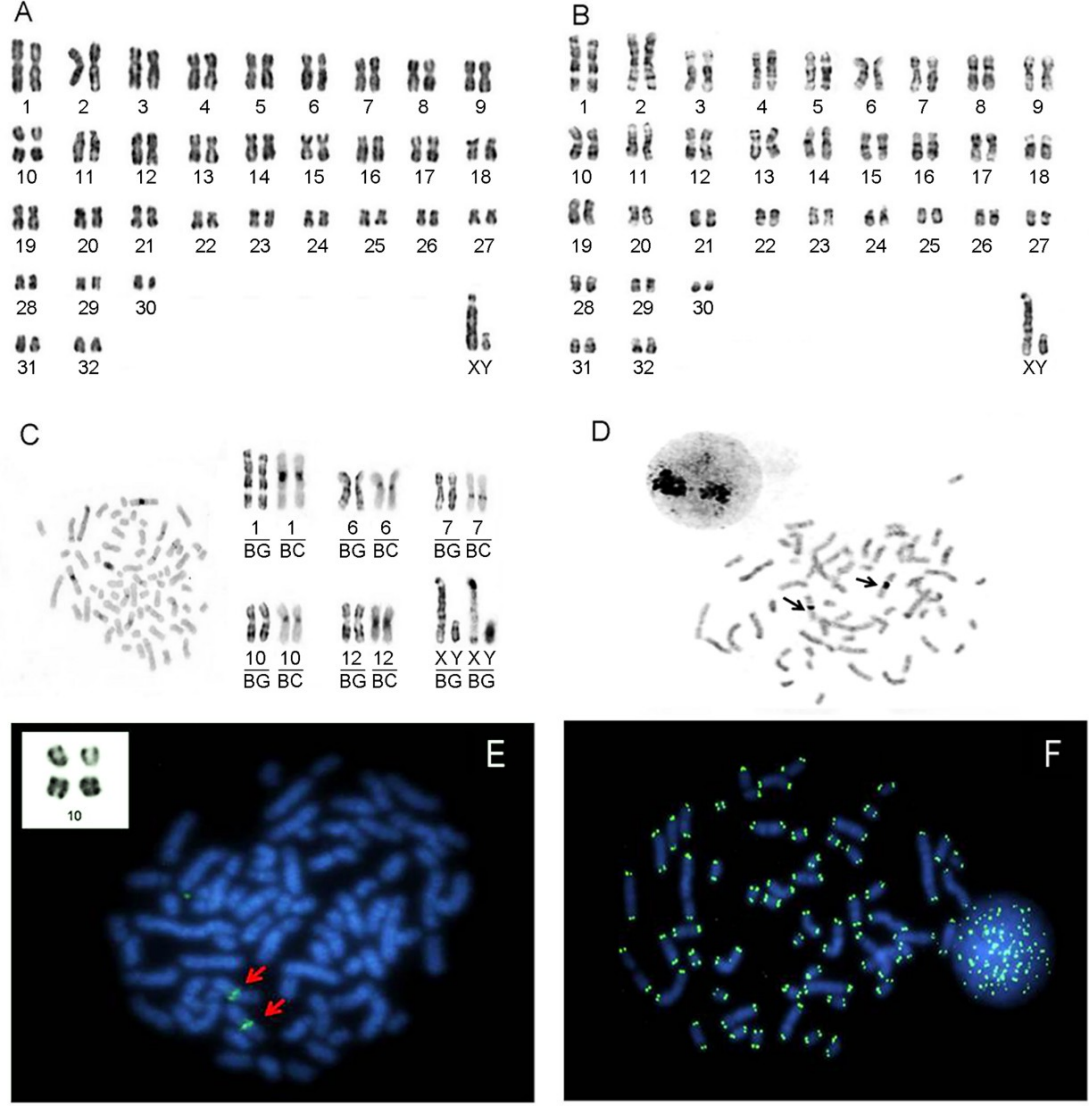
Karyotype of *Makalata* sp. 5 with 2n = 66/FN = 124 (Abaetetuba, Pará, Brazil). A) Karyotype by Giemsa staining. B) G-banding. C) C-banding. D) Ag-NOR staining. The arrows show labeling on pair 10. E) FISH with probes for 18S rDNA sequences, labeling pair 10 (upper left side inside the white rectangle, highlighting pair 10 with evident secondary constriction). F) FISH with human telomeric sequences.

*Makalata* sp. 5 has 2n = 66/FN = 124 and 30 autosomal bi-armed pairs (meta and submetacentric) that vary gradually in size plus two small one-armed pairs (acrocentric, pairs 31 and 32). The X chromosome is a large subtelocentric and the Y chromosome is small submetacentric (Fig 2A and 2B). According to the criteria used in Table 1, this would be karyotype K.

Constitutive heterochromatin (CH) is present only in pairs 1, 6, 7, 10, 12, X and Y (Fig 2C). In pairs 6 and 10, it is present only in the centromeric region. In pairs 1 and 12, the CH occurs in the pericentromeric region. Pair 7 has a small block of CH in the proximal region of the long arm. In the X chromosome, the CH occurs in the pericentromeric region, most of the short arm, and in the terminal region of the long arm. The Y chromosome is almost all heterochromatic. The remaining pairs did not demonstrate the presence of CH (Fig 2C).

Pair 10 shows a large secondary constriction in the pericentromeric region corresponding to the Nuclear Organizing Region (NOR; Fig 2A). Ag-NO_3_ staining and FISH with 18S rDNA probes labeled the same region (Fig 2D and 2E).

FISH with telomeric sequence probes showed labeling only at the terminal regions of all chromosomes (Fig 2F).

### *Makalata didelphoides* (Porto Grande, Amapá, Brazil)

**Fig 3:**
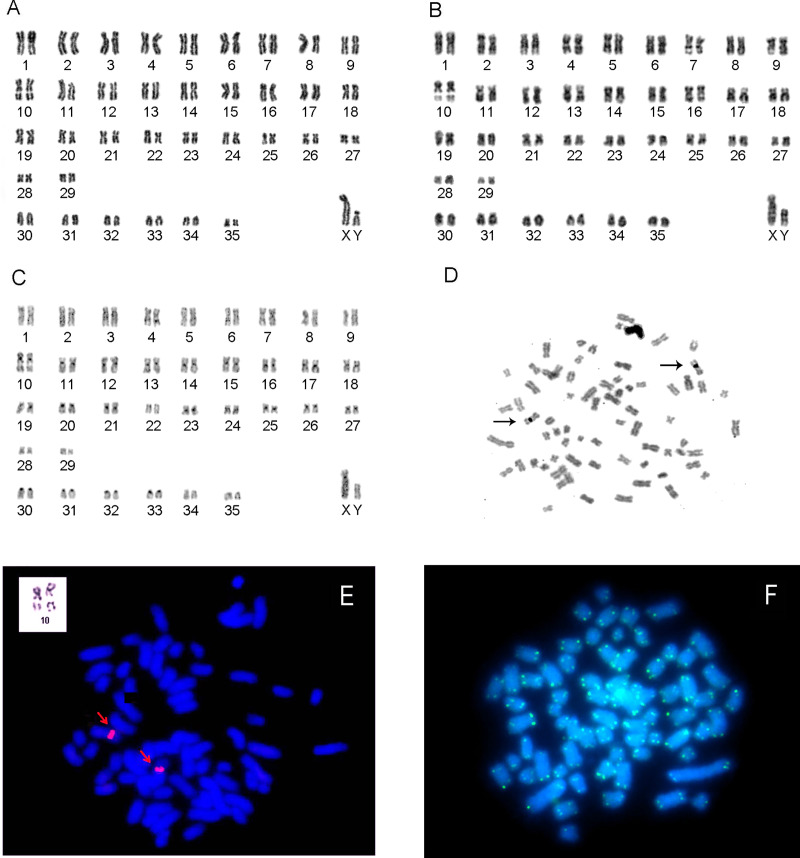
Karyotype of *Makalata didelphoides* with 2n = 72/FN = 128 (Porto Grande, Amapá, Brazil). A) Karyotype by Giemsa staining. B) G-banding. C) C-banding. D) Ag-NOR staining. The arrows show labeling of pair 10. E) FISH with probes for 18S rDNA sequences, labeling pair 10 (upper left side inside the white rectangle, highlighting pair 10 with evident secondary constriction). F) FISH with human telomeric sequences.

*Makalata didelphoides* has 2n = 72/FN = 128 and 29 autosomal bi-armed pairs (pairs 1–29, meta and submetacentric) that vary gradually in size (Fig 3A and 3B) plus six one-armed pairs (pairs 30–35, acrocentric; Fig 3A and 3B). The X chromosome is a large submetacentric and the Y is a medium-size submetacentric (Fig 3A and 3B). According to the criteria used in Table 1, this would be karyotype L.

CH is present in the centromeric regions of pairs 3, 10–21, 23–27, 30–33 and the X (Fig 3C). Pair 4 has a small block of CH in the medial region of the long arm. The other autosomes and the Y chromosome did not demonstrate the presence of CH.

Pair 10 shows a secondary constriction in the medial region of the long arm corresponding to the NOR (Fig 3A). Ag-NO_3_ staining and FISH with 18S rDNA probes labeled the same region (Fig 3D and 3E).

FISH with telomeric sequence probes demonstrated labeling only at the terminal regions of all chromosomes (Fig 3F).

## Discussion

**Fig 4:**
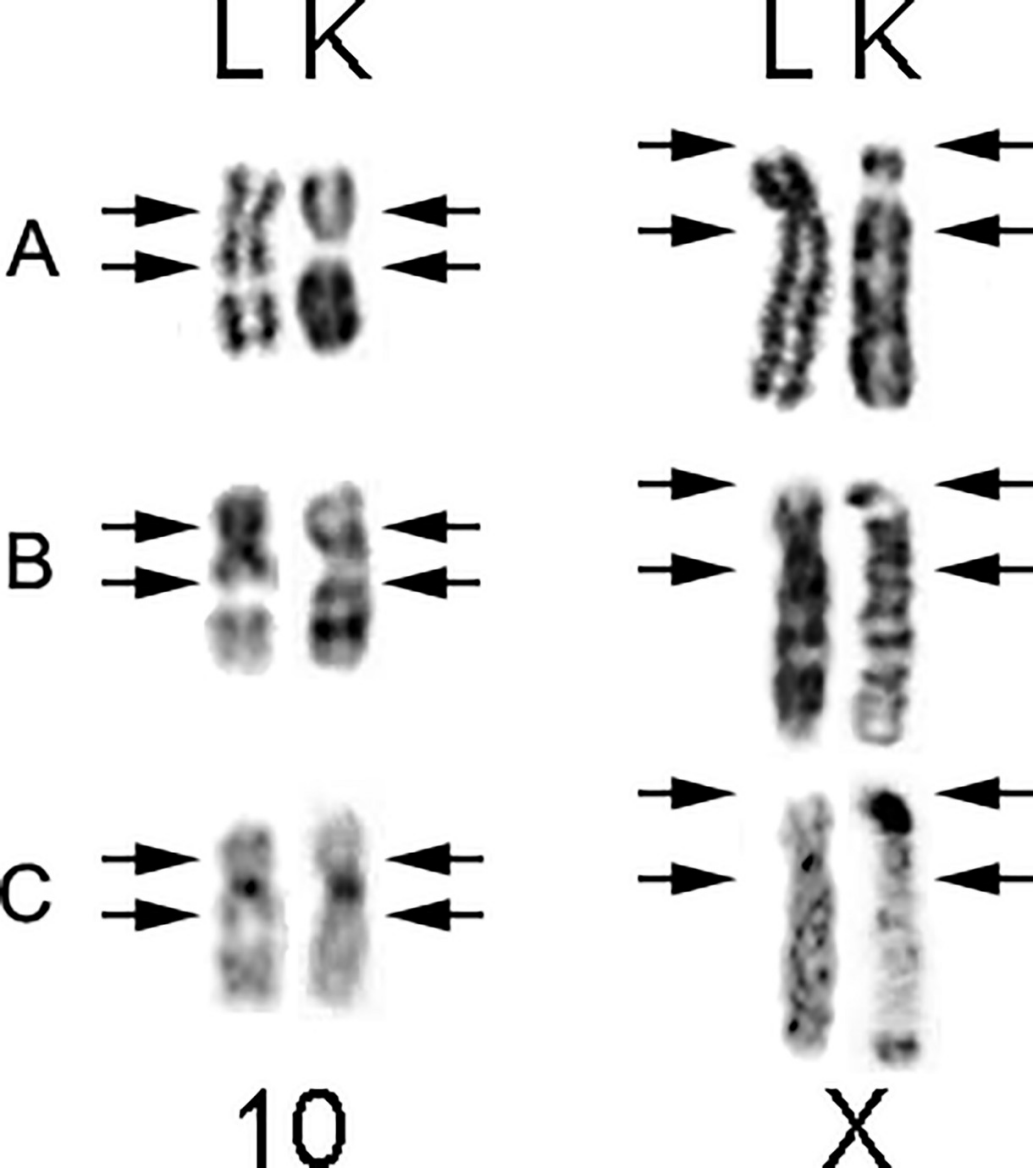
**Comparative analysis of the NOR-bearing pair (pair 10) and sex chromosomes between karyotype K (right column) and karyotype L (left column).** A) Giemsa staining. B) G-banding. C) C-banding.

The karyotype observed in *Makalata* sp. 5 (2n = 66/FN = 124, karyotype K) differs significantly from that of *M*. *didelphoides* (2n = 72/FN = 128, karyotype L). The NOR-bearing pair in karyotype K has a secondary constriction below the centromere in pair 10, whereas in karyotype L this constriction is located in the medial region of the long arm of pair 10 (Fig 4A). This difference may have originated from a paracentric inversion involving this chromosomal pair. The X chromosome in karyotype K is a large subtelocentric, while that in karyotype L is a large submetacentric (Fig 4A–4C). This difference may have originated from a pericentric inversion and addition of heterochromatin, since the short arm of X in karyotype K seems larger and is mostly heterochromatic. The Y chromosome is morphologically similar between the two species, but the one in karyotype K is almost entirely heterochromatic, whereas that in karyotype L does not have evident CH (Fig 4C). This difference may reflect CH addition/deletion events or epigenetic mechanisms leading to the formation of heterochromatin [23–25]. The difference between the diploid numbers can be attributed to fusion- and/or fission-based chromosomal rearrangements, pericentric inversions and/or centromeric transpositions [26]. Apart of this, three striking features can distinguish karyotype K from karyotype L: the NOR-bearing pair 10, the morphology of the X chromosome, and the general CH composition (Fig 4). The differences in the NOR bearing pair of the two karyotypes and the morphology of the X chromosome may reflect pericentric inversions and/or centromeric transpositions; as previously mentioned, addition/deletion events may account for the difference in the amount of CH.

*Makalata* sp. 5 (karyotype K) has the same diploid number as *M*. *didelphoides* (Karyotype A) [16], but with a different FN. We could compare them because karyotype A is unique in the literature for having chromosome banding data available. The difference in FN may be a consequence of at least nine pericentric inversions and/or centromere-repositioning events. The karyotypes also differ with regard to the content and distribution of CH. In karyotype A, the CH is restricted to the pericentromeric regions of almost all of the chromosome pairs (except in pairs 2, 3 and 4) [16]. In Karyotype K, in contrast, there is less CH; it is located on only five pairs of autosomes and the sex chromosomes (pairs 1, 6, 7, 10, 12, Y and X). Thus, despite the similarity in the diploid number, the karyotypes have large differences. These karyotypic differences, together with the fact that the taxa were located in different phylogenetic branches on a cytochrome b sequences analysis [8], suggests that the collected specimens belong to two different species.

The karyotypes attributed to the same or different species of *Makalata* exhibit huge differences in their chromosome numbers and FN (Table 1). To date, the reports that have included chromosomal data available have attributed very divergent karyotypes to the same species of *Makalata*. Molecular studies involving mitochondrial and nuclear DNA demonstrated that there is considerable haplotype divergence among samples from different locations of this genus [4, 8–10]. Fig 1 shows the geographic location of the karyotypes reported to date (including the present study) and clearly show something that may also be deduced from the molecular information: the rivers have acted as important barriers in the process of *Makalata* speciation. For instance, the taxa on the left side of the Negro-Branco rivers (karyotypes C, D and J) have karyotypes with diploid numbers ranging from 70 to 76, while those on the right side (karyotypes A and E) have diploid numbers ranging from 64 to 66; thus, the populations of the two sides have different chromosomal evolutionary histories. Molecular studies also confirmed the importance of these rivers as geographic barriers [10]. In a recent analysis [8] the populations corresponding to karyotypes F and G (and we include K) belong to the same phylogenetic branch, while those corresponding to karyotypes H and I (and we include L) belong to another branch that is connected to the branch containing F, G and K. This is called Clade B. Clade A, in turn, is closer to the tree root and includes the populations on the West Amazonia, which have karyotype J and 2n = 70 (and probably B). It has been suggested (and we agree) that the ancestral karyotype of *Makalata* has 2n = 70 or 72 [8]. Based on their karyotypic similarity, we suggest that the populations corresponding to karyotypes A and E (on the east side of the Negro-Branco rivers) are part of another branch. Thus, the chromosomal and molecular data together suggest that the existing reports have underestimated the actual number of species.

Chromosomal rearrangements contribute to reproductive isolation, since they can significantly reduce fertility in heterozygous hybrids [27]. The chromosomal differences among *Makalata* karyotypes can act as a barrier to gene flow, reducing the fertility of hybrids originating from eventual crosses among these populations. These populations are also separated by large rivers (see Fig 1). Thus, the chromosomal rearrangements and geographic barriers are likely to reinforce the reproductive isolation among the populations of *Makalata* belonging to the Brazilian states of Amazonas, Amapá, Pará and Rondonia.

## Conclusion

We herein describe two new karyotypes that increase the karyotypic diversity described for the genus *Makalata* and the Echimyidae family. The cytogenetic data reported here corroborate published molecular data suggesting that this genus contains more species than currently recognized. We believe that many of the karyotypes described to date belong to different species. Future work is needed to generate more detailed descriptions of these karyotypes, emphasizing the need for new cytogenetic studies that may contribute to our understanding of the karyotypic and taxonomic diversity of this important group of rodents.

## Supporting information

S1 Table Identification of *Makalata* species collected in Amapá and Pará States, Brazil. (DOC)
