# Supplementary material for: Karyotypic diversity within the genus Makalata (Echimyidae: Echimyinae) of Brazilian Amazon: Chromosomal evidence for multiple species
Source: PLoS One. 2020 Jul 7;15(7):e0235788. doi: 10.1371/journal.pone.0235788 (PMC7340305; doi:10.1371/journal.pone.0235788)
Supplement: S1 Table — (DOC) [file pone.0235788.s001.doc]

S1 Table. Identification of *Makalata* species collected in Amapá and Pará States, Brazil.

| **Voucher Numbers** | **Species** | **2n** | **FN** | **Sex** | **City/State** | **Geographic Coordinate** |
| --- | --- | --- | --- | --- | --- | --- |
| ABT - 01 | *Makalata* sp. | 66 | 124 | M | Abaetetuba/PA^a^ | 1°42’53.50”S; 48°54’37.90”W |
| ABT - 02 | *Makalata* sp. | 66 | 124 | F | Abaetetuba/PA^a^ | 1°42’53.50”S; 48°54’37.90”W |
| ABT - 03 | *Makalata* sp. | 66 | 124 | M | Abaetetuba/PA^b^ | 1°37’23.49”S; 48°55’33.00”W |
| PG - 01 | *M. didelphoides* | 72 | 128 | F | Porto Grande/AP | 0°42’56.60”N; 51°25’17.50”W |
| PG - 02 | *M. didelphoides* | 72 | 128 | M | Porto Grande/AP | 0°42’56.60”N; 51°25’17.50”W |
| PG - 03 | *M. didelphoides* | 72 | 128 | M | Porto Grande /AP | 0°42’56.60”N; 51°25’17.50”W |
| PG - 04 | *M. didelphoides* | 72 | 128 | F | Porto Grande/AP | 0°42’56.60”N; 51°25’17.50”W |
| PG - 05 | *M. didelphoides* | 72 | 128 | F | Porto Grande/AP | 0°42’56.60”N; 51°25’17.50”W |
| PG - 06 | *M. didelphoides* | 72 | 128 | F | Porto Grande/AP | 0°42’56.60”N; 51°25’17.50”W |
| PG - 07 | *M. didelphoides* | 72 | 128 | F | Porto Grande/AP | 0°42’56.60”N; 51°25’17.50”W |
| PG - 08 | *M. didelphoides* | 72 | 128 | M | Porto Grande/AP | 0°42’56.60”N; 51°25’17.50”W |
| PG - 09 | *M. didelphoides* | 72 | 128 | M | Porto Grande/AP | 0°42’56.60”N; 51°25’17.50”W |
| PG - 10 | *M. didelphoides* | 72 | 128 | M | Porto Grande/AP | 0°42’56.60”N; 51°25’17.50”W |
| PG - 11 | *M. didelphoides* | 72 | 128 | M | Porto Grande/AP | 0°42’56.60”N; 51°25’17.50”W |
| PG - 12 | *M. didelphoides* | 72 | 128 | F | Porto Grande/AP | 0°42’56.60”N; 51°25’17.50”W |
| PG - 13 | *M. didelphoides* | 72 | 128 | F | Porto Grande/AP | 0°42’56.60”N; 51°25’17.50”W |

(2n) Diploid Number; (FN) Fundamental Number; (PA) Pará State; (AP) Amapá State. (a) Specimens captured on the banks of Sirituba River, Abaetetuba Town. (b) Specimens captured on the banks of Caripetuba River, Abaetetuba Town.
